# Spanish media coverage of youth mental health issues during the COVID-19 pandemic

**DOI:** 10.1192/j.eurpsy.2023.94

**Published:** 2023-07-19

**Authors:** J. P. Carrasco Picazo

**Affiliations:** Psychiatry, Hospital Clínico Universitario de Valencia, Valencia, Spain

## Abstract

**Abstract: Introduction:**

The media portrayal of mental health is relevant in shaping the population’s attitudes towards mental disorders. However, there is little information about the representation of children and adolescent mental health in the Spanish press, especially in the context of the COVID-19 pandemic. The general objective of this study was to analyze the tone and content of Spanish newspaper articles about mental disorders in youth during the COVID-19 pandemic.

**Method:**

We collected media articles from 10 news sources, comprising the digital editions of five online news websites and five printed newspapers over a 6 month period (January 2021-June 2021). These articles were coded for content using a standardized codebook, followed by a qualitative thematic analysis. A total of 205 news items were evaluated.

**Results:**

Results showed that the majority of the news items had an overall positive tone (68.3%), only 5.4% were stigmatizing and only 7.3% were related to violence. However, few articles offered help seeking information (6%), adolescents were rarely quoted (14%) and children were never quoted. Substantial differences are described in terms of age, gender and disorder, with adolescents, males and patients with psychosis or behavioral disorders most associated with stigmatizing content or violence. The thematic analysis led to three emergent themes: (i) violence and victimization; (ii) the COVID-19 pandemic; and (iii) technology and social media. The number of articles that described young people with mental health problems as victims of violence was prominent.

**Conclusions:**

The Spanish media generally does not stigmatize mental health problems in children and adolescents. Furthermore, the COVID-19 pandemic may have promoted more positive discussion about youth mental health. However, there remains some room for improvement, as patients are seldom quoted, very few articles offer help-seeking information, and a narrative of victimization without appropriate discussion of resilience regularly occurs.

**Disclosure of Interest:**

None Declared

